# Supplementary figures and images for: Predictive immunoinformatics reveal promising safety and anti-onchocerciasis protective immune response profiles to vaccine candidates (Ov-RAL-2 and Ov-103) in anticipation of phase I clinical trials
Source: PLoS One. 2024 Oct 21;19(10):e0312315. doi: 10.1371/journal.pone.0312315 (PMC11493244; doi:10.1371/journal.pone.0312315)

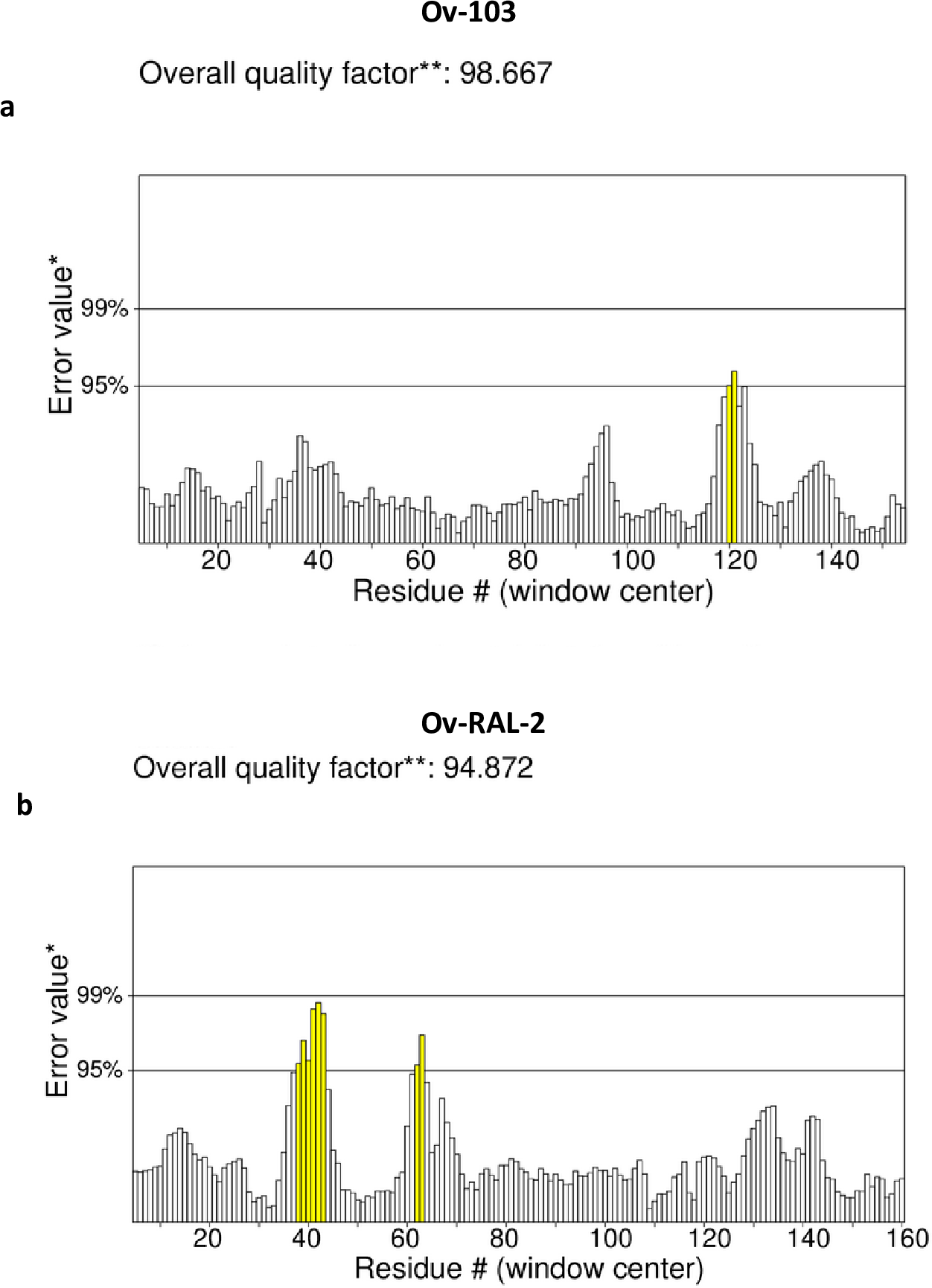

Supplement: S1 Fig — Overall template-based structural modeling quality factor of a) Ov-103 and b) Ov-RAL-2. (TIF) [file pone.0312315.s001.tif]

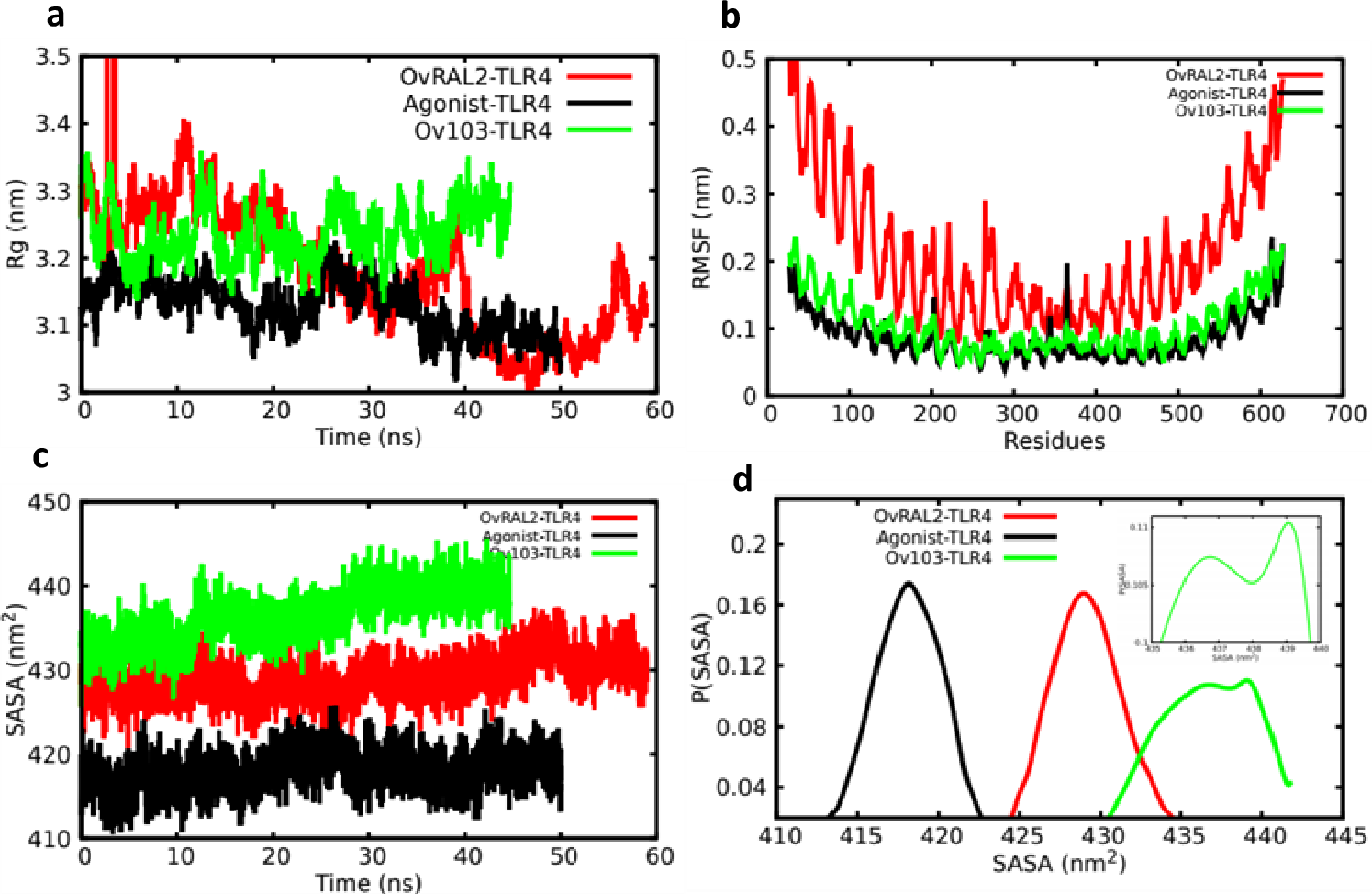

Supplement: S2 Fig — Molecular dynamics simulation evaluations of a) Radius of gyration (compactness) of antigens bound to TLR4 receptor b) Root mean square fluctuation (RMSF) c) Solvent accessible surface area (SASA) and d) Probabilities of SASA deviations. (TIF) [file pone.0312315.s002.tif]
