# Supplementary material for: Predictive immunoinformatics reveal promising safety and anti-onchocerciasis protective immune response profiles to vaccine candidates (Ov-RAL-2 and Ov-103) in anticipation of phase I clinical trials
Source: PLoS One. 2024 Oct 21;19(10):e0312315. doi: 10.1371/journal.pone.0312315 (PMC11493244; doi:10.1371/journal.pone.0312315)
Supplement: Attachment — Submitted filename: Comments-Ovolvulus.pdf [file pone.0312315.s006.pdf]

The study adds to the existing evidence of safe and protective immune response generation by the Ov-RAL-2 and Ov-103 vaccine candidates for human protection against onchocerciasis. This immunoinformatics based profiling of these two potential vaccine candidates provides significant information in anticipation of phase I clinical trials.

Comments:

1. Antigenicity of both Ov-RAL-2 and Ov-103 were not found significant when tested by ANTIGENpro, and antigenicity score of both antigens by VaxiJen was also found on the borderline of significance cutoff (0.5). Can the authors support this data with some evidence from literature where significance estimated by these tools is lesser but the antigens showed good response (antigenicity) in clinical trials. The authors can also use an additional tool to find if antigenicity is significant or not.
2. While this study is focused to characterize the profiles of each candidate individually, it will add to the study if the authors can also comment about profiling for multi-epitope vaccine design including the epitopes from both the antigens in the discussion, and cite the relevant study from existing literature about co-administration of the two proteins for generation of durable protective immune response against *O. volvulus* (PMID: 37515028).
3. One of the strong challenges identified from this study is the observed contrast between immune simulation and binding affinity and the authors have well stated that a more comprehensive evaluation of the vaccine candidate interactions with other immune receptors in addition to TLR4 can provide a more complete picture of their efficacy. The authors should mention here some of the other immune receptors (or receptor complex proteins) from the literature that should be tested in future for their binding efficacy to the antigens.
4. I suggest the authors to move plot legends (for example Fig 2c and 2d) in the empty space (below the inset plots) so that both plots and legends are clearer.
5. Figure 5, c) is missing in legend
6. References 82 and 86 are same. Authors should re-check all the references and their respective citations in the manuscript before resubmission.
